# Adaptation of Hansen solubility parameter in evaluating transparency of composite materials

**DOI:** 10.1016/j.heliyon.2019.e02833

**Published:** 2019-12-12

**Authors:** Nobuyuki Fujiwara, Takahiro Nishida, Hideki Yamamoto

**Affiliations:** Department of Chemical, Energy and Environmental Engineering, Faculty of Environmental and Urban Engineering, Kansai University, Japan

**Keywords:** Materials chemistry, Hansen solubility parameter, Organic solvent, Transparency

## Abstract

In the preparation of polymer-based functional materials, it is often difficult to express the desired function using a single substance. Thus, multiple materials are often combined to achieve a desired function using methods such as the addition of a filler or lamination. However, when materials are mixed using a filler, the transparency of the polymer decreases. Therefore, a prediction indicator for transparency is needed. In this study, we focused on using the Hansen solubility parameter (HSP) as a predictor of transparency. The value of *δ*_*d*_, which is the dispersion force term of the solubility parameter, is considered to be related to the refractive index of the solvent. Silica particles were selected as model particles, and the HSP value was determined. We examined the possibility of evaluating the transparency in a solvent containing silica particles based on the HSP value, and our results indicated that a smaller difference in *δ*_*d*_ between the particles and solvent corresponded with a higher transparency. The HSP value could be used as an index for evaluation of the dispersibility and solubility of the polymer. By using HSP theory in the material design of composite materials, it is thus considered possible to use the same index to simultaneously evaluate the dispersibility evaluation and predict the transparency of the filler.

## 1 Introduction

Recently, plastic films have attracted attention as a potential substitute for glass as a display material. Plastic films have mechanical advantages such as thinness and breakage resistance compared with glass. The properties generally desired for plastic films include high heat resistance, transparency, and chemical resistance [1]. However, it is difficult to achieve these desired functions using a single resin. To develop multiple functionalities, materials must be combined [1–5]. Common processing methods include the addition of inorganic materials [1,2], fiber reinforcement [3,6] and lamination [4,7]. Examples of typical fillers used include silica and TiO_2_. The addition of a filler to the resin often improves the heat resistance and mechanical strength [1,2,8]; however, the transparency may be impaired. It is thus necessary to predict whether transparency will be lost when the filler is added to the resin, and there is an urgent need for indicators that can accurately predict transparency when multiple materials are mixed. A method for predicting the transparency of a single resin from the molecular structure has previously been reported [9]. However, no prediction method for the transparency after the addition of a filler to solvents and resins has been reported. The resulting transparency upon addition of the filler is unknown until it is tested. Therefore, in this study, we focused on using the Hansen solubility parameter (HSP) as a predictor of transparency [5,10]. The Hildebrand solubility parameter (*δ*_*t*_) [11] is commonly used to evaluate the cohesion energies of substances. The solubility parameter, a physical property representing the cohesion energy density of a substance, is useful for evaluating the compatibility, wettability, and cohesiveness or dispersibility of substances. Hansen further refined the Hildebrand solubility parameter as consisting of three components based on the type of molecular interaction involved: namely, dispersion forces (*δ*_*d*_), intermolecular dipole interactions (*δ*_*p*_), and hydrogen-bonding interactions (*δ*_*h*_) [10,12].

The HSP is currently used to evaluate the affinity between substances such as the dispersibility of fine particles [5,13] and solubility of various substances [10]. In addition, the HSP has recently been used for a wide range of applications such as to evaluate the adsorption amount [14] or membrane separation [15].

One example of application of the HSP theory to the composite material field is the selection of gelation agent [16]. Each term of the HSP has been reported to correlate with a physical property of the solvent [12]. Hildebrand's solubility parameter *δ*_*t*_ has been reported to correlate with the surface tension [11,12]. *δ*_*d*_ and *δ*_*p*_ have been reported to be related to the refractive index and dielectric constant, respectively [12]. In addition, Novaki et al. recently reported that a correlation between the value of a Lewis acid base that can be measured by solvatochromism and *δ*_*h*_ [17]. In addition to using the HSP as an index to evaluate and predict the transparency of composite materials, it will also be possible to select the optimum dispersion medium and surface treatment agent for the filler [5]. HSP theory is considered a more capable tool than group contribution methods for material design [9]. In this study, we focused on *δ*_*d*_, which is related to the refractive index, and examined the transparency of solid–liquid and solid–solid systems. Spherical silica, which is generally used as a filler, was used as a model substance. The HSP value of the silica was determined, and the silica particles were dispersed in a solvent of known HSP. The transparency was determined by examining the light transmission in various dispersion media. For the solid–solid system, the spherical silica was dispersed in a polymer to make a polymer sheet, and the light transmittance of the sheet was measured. The relationship between the light transmittance and the difference between the *δ*_*d*_ term of silica and the dispersion medium was determined.

## 2 Theory

### 2.1 Theory of Hansen solubility parameter

The solubility parameter *δ*_*t*_ [(MPa)^1/2^] used in the solubility evaluation was defined as

(1) δt=(ΔEVVM)12

where ΔE^V^ is the liquid cohesion energy [J] and V_M_ is the molar volume [cm^3^/mol] [11].

Hansen divided the cohesion energy ΔE^V^ [J] of the Hildebrand solubility parameter into three components (i.e., dispersion interactions ΔE^V^_d_ [J/mol], dipole interactions ΔE^V^_p_ [J/mol], and hydrogen-bonding interaction ΔE^V^_h_ [J/mol]), which can be expressed as follows [10,12,14]:

(2) $$\Delta E^{V}=\Delta E_{d}^{V}+\Delta E_{p}^{V}+\Delta E_{h}^{V}$$

(3) δd=(ΔEdVVM)12,δp=(ΔEpVVM)12,δh=(ΔEhVVM)12

(4) $$\delta_{t}^{2}=\delta_{d}^{2}+\delta_{p}^{2}+\delta_{h}^{2}$$

where, *δ*_*d*_ [(MPa) ^1/2^], *δ*_*p*_ [(MPa) ^1/2^] and *δ*_*h*_ [(MPa) ^1/2^] represent the dispersion force factor, dipole interaction force factor, and hydrogen-bonding force factor of the HSP, respectively. Quantitative evaluation of the solubility is represented by the value of *R*_*a*_ [(MPa)^1/2^], which reflects the distance between the HSPs of both substances:

(5) Ra=[4·(δd,1−δd,2)2+(δp,1−δp,2)2+(δh,1−δh,2)2]12

A smaller *R*_*a*_ means a higher solubility of each substance because the interaction forces acting between the molecules are similar. Thus, substances with large *R*_*a*_ values exhibit low solubility.

The HSP approach uses information from a database to calculate values by the group contribution method. However, metal compounds and fine particles cannot be used to determine HSP values easily [12]. Therefore, the Hansen Solubility Sphere method is used in this experiment [5,[12], [13], [14]].

Results of the affinity evaluations can be visualized by plotting the results of *δ*_*d*_, *δ*_*p*_ and *δ*_*h*_ measurements in a three-dimensional graph. When the solubility parameters of good and poor solvents for a desired substance are plotted in such a three-dimensional figure, data points for good solvents cluster in a particular region in the form of a sphere called the Hansen solubility sphere. In the solubility sphere, good solvents for a desired substance are plotted inside the sphere and poor solvents are plotted to outside of the sphere. The HSP components of the desired substance are defined by the center of the sphere [5,[12], [13], [14]]. The radius of the sphere is called the interaction radius R_0_ [(MPa) ^1/2^].

The Hansen solubility sphere model is based on the theory that dissolution is promoted when the difference in the solubility parameters is small. However, silica fine particles do not dissolve. Therefore, different affinity factors are required. In this study, we focused on wettability as a factor of affinity. If the affinity between solid and liquid is high, the solvent spreads out. On the contrary, it is an idea that if the affinity between solid and liquid is poor, it does not spread. In this study, silica was molded into a disc and the contact angles to various solvents were measured. From the measurement results, HSP of the silica particle surface was determined by the Hansen solubility sphere method.

It is reported that the *δ*_*d*_ term of HSP has a relationship with the refractive index. Hansen expressed the relation between the dispersion power term of HSP and the refractive index as follows [12].

(6) $$\delta_{d}=\frac{n_{D}-0.784}{0.0395}$$

Here, *δ*_*d*_ [(MPa) ^1/2^] represents a dispersion force term, and *n*_*D*_ [-] represents a refractive index. The coefficients in the formula are determined using 540 data points [12].

### 2.2 Light scattering

The two main theories of light scattering by fine particles are Rayleigh scattering and Mie scattering [18]. The theoretical range that can be handled by the system is determined by the χ parameter in the following equation:

(7) $$\chi =\frac{2\pi r}{\lambda }$$

Here, χ represents the particle diameter parameter [-], r represents the particle diameter [m], and λ represents the wavelength [m] of the incident light. When χ < 1, the scattering is considered to be Rayleigh scattering; when χ ≧ 1, it can be considered to be Mie scattering [19]. The particle size of the silica particles used in this study was 500 nm. The wavelength range of light studied was 350–700 nm in the visible region. Therefore, the range of χ was 4.5 ≦ χ ≦ 9.0, which is in the theoretical range of Mie scattering.

### 2.3 Scattering efficiency

The scattering efficiency, which indicates how much incident light scatters in a composite material to which filler particles are added, is expressed by the following equation [20]:

(8) $$Q_{ext}=2\rho \overset{\frac{\pi }{2}}{\underset{0}{\int }}sin\left(\rho \;cos\gamma \right)sin^{2}\gamma d\gamma$$

Here, *Q*_*ext*_ [-] is the scattering efficiency, *ρ* [-] is the phase difference of the light when passing through the particle diameter *D*_*p*_ [m] from the center of the particle, and γ [°] is the incident angle to the particle surface of the light. Also, *ρ* [-] is expressed as [19].

(9) $$\rho =\frac{2\pi }{\lambda }D_{p}\left(n_{m}-n_{p}\right)$$

Here, λ [m] is the wavelength of the incident light, *n*_*m*_ [-] is the refractive index of the dispersion medium, and *n*_*p*_ [-] is the refractive index of the particle. For a plastic film to be applied as a display body, the scattering efficiency *Q*_*ext*_ [-] must be 0.05 or less. In the formula, the physical property values that can be changed in the material design are the particle diameter and refractive index, and it is conceivable that the dispersibility and refractive index difference between the filler particles and plastic film greatly affect the transparency of the system.

## 3 Experimental section

### 3.1 Experimental materials

A special-grade reagent manufactured by Wako Pure Chemical Industries, Ltd. was used as the organic solvent, and Hypressic N2N spherical silica particles (Ube-exsymo Ltd. Tokyo, Japan) were used as model particles. A list of solvents used in this study is shown in Table S1. The primary particle diameter of the silica particles was 500 nm. In addition, in this study, the solvent set used for measuring the HSP and light transmittance differed. The solvent set used for the Hansen dissolving sphere method should be present in a wide area in Hansen's 3D graph. Experiments were conducted focusing on the value of *δ*_*d*_ in the light transmittance measurement. Therefore, the solvent set used for determining the light transmittance was selected such that the value of *δ*_*d*_ was widely dispersed in the range of 14.5–21.0 (MPa)^1/2^.

A total of three polymers were used as solid-solid dispersion media. Two types of polymers with different styrene-methacrylic acid complex ratios and Silicone-methacrylic acid composite adhesive. Because CEMEDINE C is a solvent-based adhesive, it was necessary to remove the solvent. To obtain only the polymer part, the adhesive was placed on a Teflon sheet in air for 24 h to sufficiently remove the solvent, and the polymer part was used in the experiment. In this paper, the polymer part from which the solvent has been removed is called CEMEDINE C.

### 3.2 Method of measuring HSP value of particle surface

**Figure 1:**
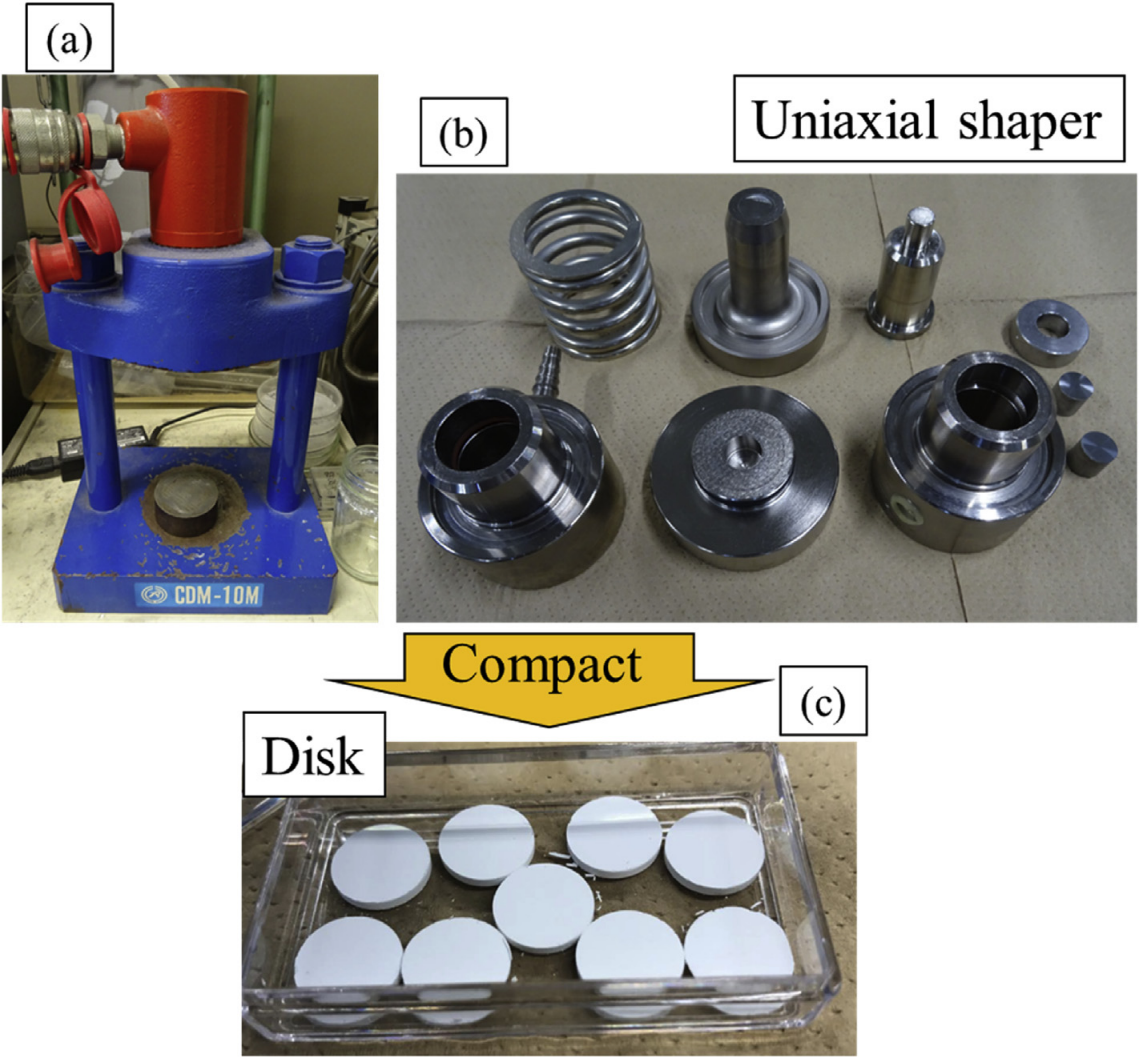
Disk molding example of silica particles. (a) Compression molding machine, (b) Mold parts, (C) Silica disk after molding.

A 5.0 wt% polyvinyl alcohol (PVA) aqueous solution was added and mixed to achieve a ratio of 0.2 mL of PVA per gram of silica particles. The PVA aqueous solution played the role of a binder. The prepared particles were dried at 353 K for 24 h and then compression molded for 1 min at a pressure of 40 MPa using a single-shaft molding machine to form a disc. A representative example of the disk-shaped silica particles is shown in Figure 1. The contact angle measurement was performed on 17 types of organic solvents using a contact angle measurement device (Kyowa Interface Science Co., Ltd. DMs-400, Tokyo Japan). The amount of solvent to be dropped was fixed at 4 μL. The contact angle 100 ms after dropping the droplet was measured three times using the same solvent, and the average value was adopted.

In order to obtain the HSP of the particle surface, the contact angle was measured using the target particle, not the glass slide. The amount of PVA added is negligible. Therefore, it is considered that the obtained HSP value can ignore the influence of PVA.

### 3.3 Method of measuring HSP value of polymers

20 mL of an organic solvent was added to 0.1 g of each polymer. Shaking and stirring were performed for 24 h in a thermostatic bath in which the water temperature was set to 25 °C. After stirring, the solubility of the polymer was confirmed by visual evaluation. The HSP value was calculated by the Hansen solubility sphere method based on the result of the visual evaluation.

The reason why for the difference in the number of solvents is that when determining the HSP value by the Hansen solubility sphere method it is necessary to have a sufficient dispersion on the 3D graph with poor solvents surrounding the sphere. The interaction radius of the spheres for each material and their position on the 3D graph are different, hence, we selected different solvents.

### 3.4 Polymer film forming method

Silica particles were added to 30 mL of tetrahydrofuran (THF) and sonicated for 10 min. Various polymers were dissolved in the dispersion medium. After polymer dissolution, sonication was performed for 10 min. The solution was poured into a cell in which a Teflon sheet and a glass slide were combined and heated on a hot plate for 2 h to remove THF. A schematic diagram of the preparation is presented in Figure S1. In addition, the optimum conditions for the polymer addition amount, particle addition amount, and heating temperature at the time of film formation were examined. Two out of the three conditions of polymer addition amount, particle addition amount, and heating temperature were fixed, and the optimum condition of each condition was searched. The polymer concentration was examined under conditions of 5.0, 1.0 and 0.5 g for 30 ml of THF. The heating temperature was examined at 40, 50 and 60 °C. The amount of added particles was examined under the conditions of 0.1, 0.01 and 0.001 g for 30 ml of THF. In each fixing condition, the polymer concentration was 5.0 g, the particle addition amount was 0.01 g, and the heating temperature was 60 °C. The preparation conditions were varied for each polymer. A total of 21 types of films were prepared. Table S2 shows the conditions of the film created in this study.

If unevenness is present on the adjusted film surface, it may affect the transmission measurement result and led to error. To evaluate the degree of this “non-uniformity”, the thickness at 10 points on the film was measured with a micrometer (Mitutoyo Corporation, M110, OM), and the average value and standard deviation were calculated. The range of the error in the measurement result resulting from the uneven thickness was confirmed.

### 3.5 Transmittance measurement

The particles were added to the organic solvent to achieve a concentration of 5.0 × 10^−4^ g/mL. The particles were dispersed in the solvent using ultrasonic dispersion for 5 min. The transmittance of light in various dispersion media was measured using a visible spectrophotometer (ASONE Co., ASUV-6300PC). The solvent was selected such to achieve a wide range of the *δ*_*d*_ term of the HSP. In this study, wavelengths of 300–900 nm were measured. The light transmittance after particle addition was examined in the visible region of 350–700 nm. However, solvents with a specific peak at approximately 350 nm, such as N-methyl aniline, were examined excluding that part. In addition, to examine the relationship between the transmittance and particle size in the solvent, the particle size in the solvent was measured using a concentrated particle size analyzer (Otsuka Electronics Co., Ltd., FPAR-1000). The solvent used for the measurement was the same as that used for the light transmittance measurement. When the particle size was measured, each sample was subjected to ultrasonic treatment for 5 min to ensure that the particles were in a dispersed state. Measurement conditions such as the weight of particles added to the solvent are based on the author's previous paper [5]. The film with added particles was measured for scattering intensity and transmittance. A 9 × 33 mm film was applied to the inside of the measurement surface of the visible light display cell, and the scattering intensity and transmittance were measured. The measurement was performed three times. The wavelength of the laser beam emitted by the zeta potential/particle size/molecular weight measuring device system was 660 nm. At the time of measurement of the transmittance of visible light, background correction was performed using a polymer film with no fine particles added.

## 4 Results and discussion

### 4.1 HSP measurements of silica particle surface and polymer

**Figure 2:**
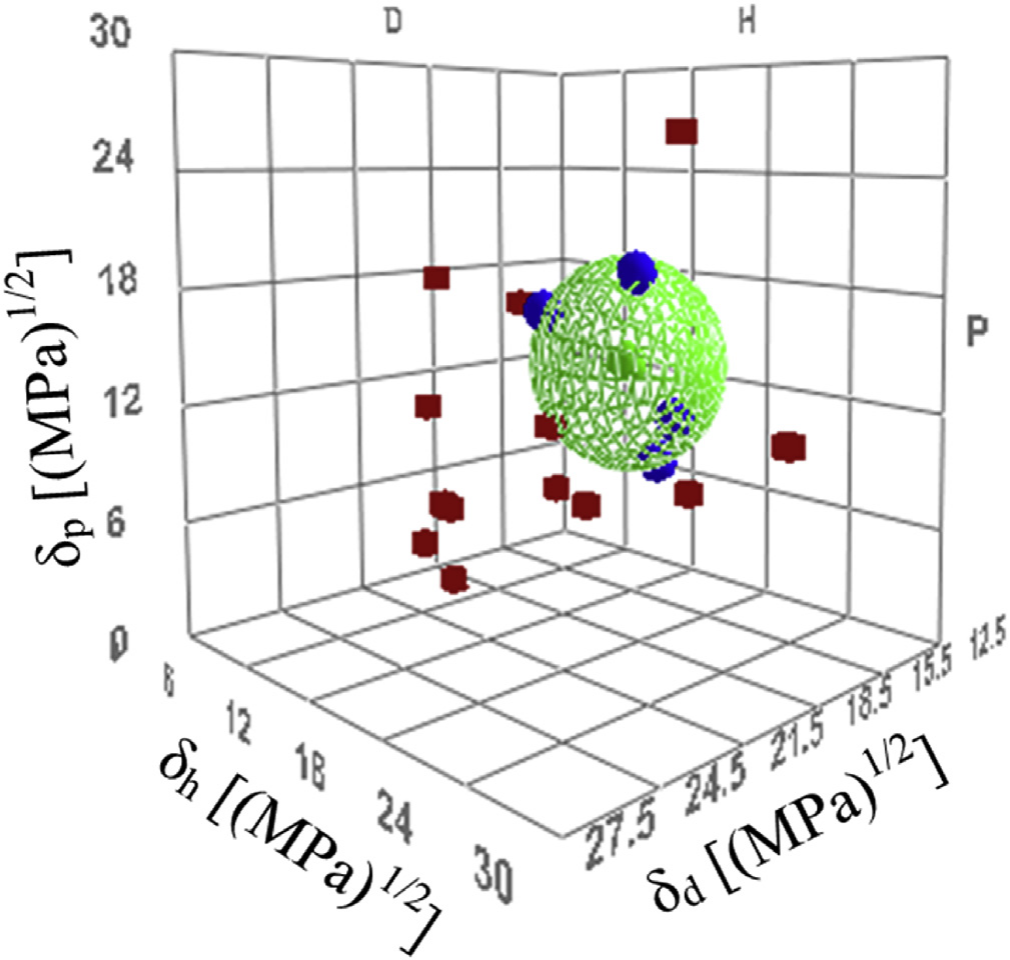
HSP 3D diagram of Silica particles with HSPs of organic solvents used in this work. Blue balls are “good” solvents, and red cubes are “poor” solvents.

Table 1 presents the measurements of the contact angle of each solvent to the disc-shaped particles as well as the HSP and surface tension of the solvent [21]. The contact angle was 26.0° in 1-propanol. In addition, the contact angle was the smallest in N-methyl formamide, which has a relatively high surface tension. These results indicate that the affinity between the solid surface and liquid and not the effect of the surface tension was an important factor, with a difference appearing in the contact angle. Thus, the contact angle measurement was effective for evaluation of the affinity. The Hansen solubility sphere created from the results in Table 2 is shown in Figure 2. The HSP values of the silica particle surface determined from the Hansen solubility sphere were *δ*_*d*_ = 16.7 [(MPa)^1/2^], *δ*_*p*_ = 13.7 [(MPa)^1/2^], and *δ*_*h*_ = 14.0 [(MPa)^1/2^].

**Table 1:** Contact angle of each solvent to disc and HSP value and surface tension of solvents. (A score of 1 indicates a good solvent and a score of 0 indicates a poor solvent.)

Solvent	*δ*_*d*_ [(MPa)^1/2^]	*δ*_*p*_ [(MPa)^1/2^]	*δ*_*h*_ [(MPa)^1/2^]	Surface tension (25 °C) [mN/m]	Contact angle [°]	Score [-]
N-Methyl formamide	17.4	18.8	15.9	39.58	13.6	1
Allyl alcohol	16.2	10.8	16.8	25.38	14.7	1
Dimethyl sulfoxide	18.4	16.4	10.2	42.92	15.3	1
Ethylene glycol monomethyl ether	16.0	8.2	15.0	30.84	16.0	1
Propylenecarbonate	20.0	18.0	4.1	42.00	17.1	0
γ-Butyrolactone	18.0	16.6	7.4	43.44	17.4	0
Formamide	17.2	26.2	19.0	57.02	17.7	0
Salicy aldehyde	19.0	10.5	12.0	42.28	18.1	0
1-Bromonaphthalene	20.6	3.1	4.1	43.90	18.2	0
Tetrahydrofuran	16.8	5.7	8.0	26.50	19.0	0
1-Methyl naphthalene	19.7	0.8	4.7	37.63	19.1	0
Nitrobenzene	20.0	10.6	3.1	42.00	19.7	0
Quinoline	20.5	5.6	5.7	42.59	21.7	0
Benzyl alcohol	18.4	6.3	13.7	35.97	24.0	0
1-propanol	16.0	6.8	17.4	23.31	26.0	0
Benzyl benzoate	20.0	5.1	5.2	45.41	26.7	0
Ethylene Glycol	17.0	11.0	26.0	47.99	27.9	0

**Table 2:** Solubility score of polymers in select organic solvents and HSPs of used organic solvents. (A score of 1 indicates a good solvent and a score of 0 indicates a poor solvent; –: not measured.)

Solvent	*δ*_*d*_ [(MPa)^1/2^]	*δ*_*p*_ [(MPa)^1/2^]	*δ*_*h*_ [(MPa)^1/2^]	Score		
				S-A	S–B	CEMEDINE-C
1,4-Dioxane	17.5	1.8	9.0	0	0	0
1-Butanol	16.0	5.7	15.8	0	0	0
Acetone	15.5	10.4	7.0	0	0	1
Acetonitrile	15.3	18.0	6.1	0	0	1
Carbon Disulfide	20.2	0.0	0.6	0	1	-
Chloroform	17.8	3.1	5.7	1	1	0
Dimethyl Sulfoxide	18.4	16.4	10.2	0	0	-
Ethyl Acetate	15.8	5.3	7.2	0	0	1
Methyl Ethyl Ketone	16.0	9.0	5.1	1	0	1
Methyl Isobutyl Ketone	15.3	6.1	4.1	0	0	1
Nitrobenzene	20.0	10.6	3.1	0	0	0
N-Methyl-2-Pyrrolidone	18.0	12.3	7.2	0	0	1
Octane	15.5	0.0	0.0	0	0	-
Pyridine	19.0	8.8	5.9	0	1	1
Quinoline	20.5	5.6	5.7	0	0	-
Tetrahydrofuran	16.8	5.7	8.0	1	1	1
γ-Butyrolactone	18.0	16.6	7.4	0	0	1
Dimethyl Formamide	17.4	13.7	11.3	-	-	1
N-Methyl Formamide	17.4	18.8	15.9	-	-	1
Cyclohexane	16.8	0.0	0.2	-	-	0
Toluene	18.0	1.4	2.0	-	-	0
Ethanol	15.8	8.8	19.4	-	-	0
Ethylene Glycol Monobutyl Ether	16.0	5.1	12.3	-	-	0
1,1,2,2-Tetrabromoethane	21.0	7.0	8.2	-	-	0
Allyl Alcohol	16.2	10.8	16.8	-	-	0
Formamide	17.2	26.2	19.0	-	-	0
Ethanolamine	17.0	15.5	21.0	-	-	0

**Figure 3:**
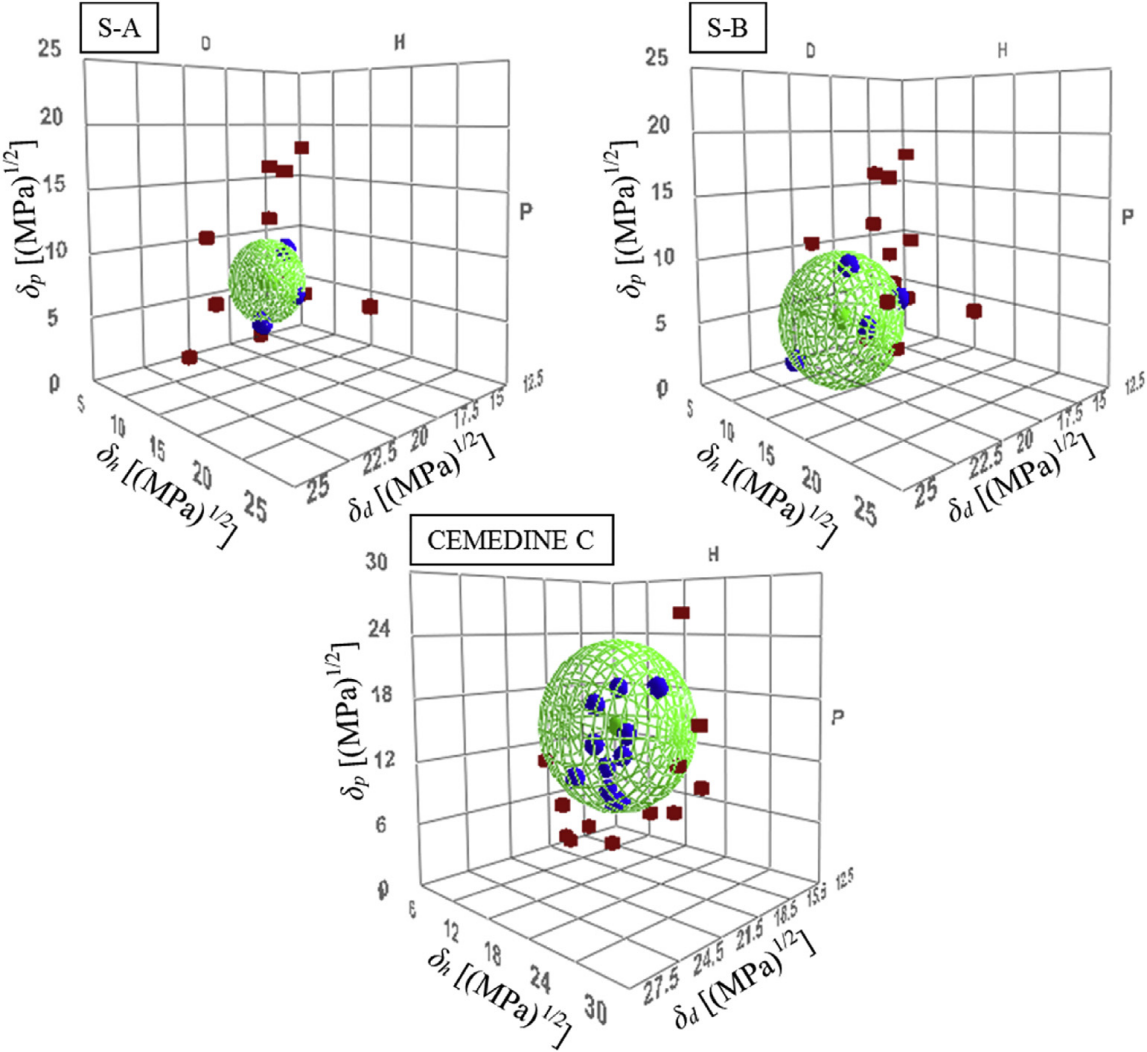
HSP 3D diagram of each resins with HSPs of organic solvents used in this work. Blue balls are “good” solvents, and red cubes are the “poor” solvents: (S–A) Hansen solubility sphere of S-A, (S–B) Hansen solubility sphere of S–B (CEMEDINE C) Hansen solubility sphere of CEMEDINE C.

Table 3 presents the evaluation results of the dissolution test for each polymer. In addition, the Hansen sphere and HSP values determined from the results in Table 3 are presented in Figure 3 and Table 4. The smallest difference from the value of *δ*_*d*_ of the silica particles was observed for CEMEDINE C. That is, when CEMEDINE C was selected as the dispersion medium, the light transmittance was expected to be the highest.

**Table 3:** HSPs of dispersion medium polymers.

material	*δ*_*d*_ [(MPa)^1/2^]	*δ*_*p*_ [(MPa)^1/2^]	*δ*_*h*_ [(MPa)^1/2^]	*δ*_*t*_ [(MPa)^1/2^]
S-A	17.2	6.4	5.2	19.1
S–B	18.5	3.7	3.7	19.2
CEMEDINE C	16.5	14.1	8.1	23.2
Model particle	16.7	13.7	14.0	25.7

**Table 4:** Results of dispersibility and light transmittance experiment at wave length = 400, 500, 600 and 700 [nm].

Solvent	*δ*_*d*_ [(MPa)^1/2^]	*δ*_*p*_ [(MPa)^1/2^]	*δ*_*h*_ [(MPa)^1/2^]	Wave length [nm]				Particle size [nm]
				400	500	600	700	
Acetic Acid	14.5	8.0	13.5	25.8	37.3	48.2	57.2	878.7
Acetone	15.5	10.4	7.0	26.5	37.4	47.6	55.8	684.7
Acetonitrile	15.3	18.0	6.1	11.4	20.2	30.4	39.9	446.4
Aniline	20.1	5.8	11.2	1.1	4.4	10.4	18.0	526.7
Cyclohexanone	17.8	8.4	5.1	81.3	89.2	92.9	95.2	579.1
Diethylene Glycol Monomethyl Ether	16.2	7.8	12.6	95.6	97.0	97.7	98.2	593.0
Dimethyl Sulfoxide	18.4	16.4	10.2	41.3	59.9	71.0	77.9	711.7
Dipropylene Glycol	16.5	10.6	17.7	92.3	95.6	97.3	98.1	535.8
Ethanol	15.8	8.8	19.4	26.2	38.8	50.5	59.9	570.1
Formamide	17.2	26.2	19.0	77.6	89.7	94.2	96.2	655.8
Methanol	14.7	12.3	22.3	6.6	13.8	23.5	33.4	553.1
Methyl Isobutyl Ketone	15.3	6.1	4.1	69.0	76.0	81.8	85.9	685.3
Pyridine	19.0	8.8	5.9	13.0	31.5	47.1	58.7	597.7
1,1,2,2-Tetrabromoethane	21.0	7.0	8.2	0.4	1.3	3.2	6.2	1157.5
Tetrahydrofuran	16.8	5.7	8.0	81.1	85.5	89.1	91.5	814.5
N-Methylaniline	19.5	6.0	7.8	0.0	5.0	12.2	20.8	603.4

### 4.2 Polymer film preparation conditions

Representative examples of the visual evaluation of the polymer under each preparation condition are shown in Figure S2. When the amount of polymer was changed, a sufficient thickness was obtained for polymer amounts of 1.0 and 5.0 g; however, a film was not formed when the polymer amount was 0.5 g. The uneven distribution of particles was confirmed to be the smallest at 5.0 g with a partial uneven distribution at 1.0 g. It is considered that the amount of model particles per polymer volume increases and dispersion failure or aggregation is likely to occur. Further, the thickness unevenness was large at 1.0 g. The above results indicate that the use of 5.0 g of the polymer is preferable.

When the addition amount of particles was changed, the uneven distribution of particles was small at 0.001 and 0.01 g. At 0.1 g, particle aggregates were observed to float on the film surface or particles were present in the film in the form of vortices. In addition, when the particle amount was 0.001 g, a significant amount of air bubbles were generated on the film. There were relatively few air bubbles when the amount of particles was large. This finding may result from the particles suppressing the formation of bubbles when the solvent is vaporized. Based on the above results, a particle amount of 0.01 g was selected.

When a drying temperature of 50 °C was used, a film with very few air bubbles was prepared. Moreover, at 40 °C, it was difficult to control the temperature of the hot plate, and a uniform film was not obtained. At 60 °C, a large amount of bubbles were generated because of proximity to the boiling point of THF. From the above results, a drying temperature of 50 °C was selected. The above results indicate that the polymer amount of 5.0 g, particle addition amount of 0.01 g, and drying temperature of 50 °C were optimum. Therefore, a film in which 0.01 g of particles were added to 5.0 g of polymer was used in the following examination. Moreover, the weight of each film was measured after film formation, and it was confirmed that there was no weight change. Therefore, about 99.8 wt% of the film is composed of polymer, and the remaining about 0.2 wt% is particles.

The average thickness of the SA film sample was determined to be approximately 0.182 mm based on thickness measurements of each polymer film at 10 points under the set conditions. The S–B film sample was approximately 0.195mm thick. The CEMEDINE C film sample was approximately 0.183mm thick. Furthermore, a variation of approximately 8% in the thickness was confirmed. The standard deviation indicated that an error of up to approximately 10% may be present in the measurement results.

### 4.3 Transmittance measurement result in solid-liquid system

**Figure 4:**
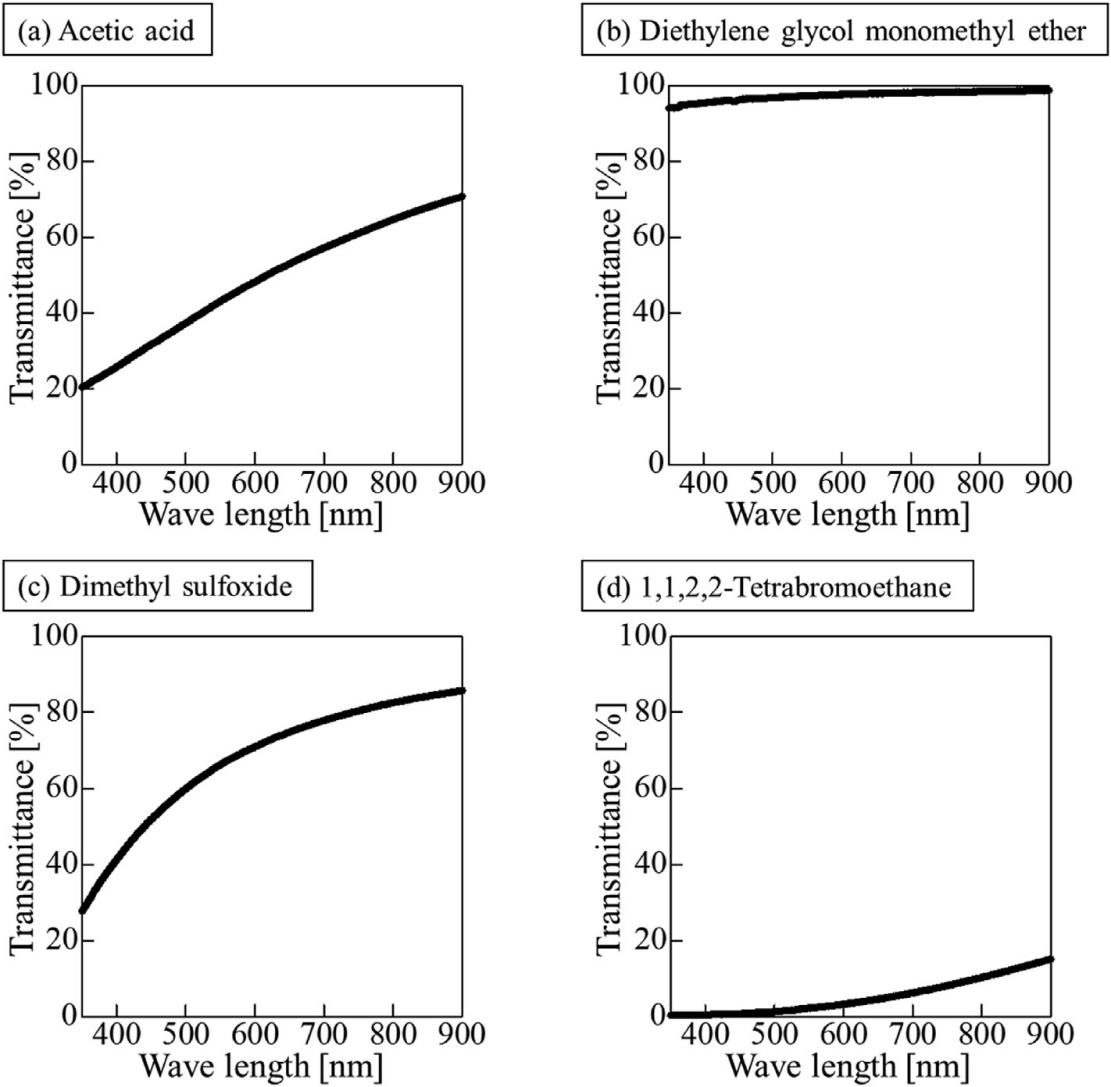
Transmittance of light in different dispersion solvents. (a)Acetic acid, (b) Diethylene glycol monomethyl ether, (c) Dimethyl sulfoxide, (d) 1,1,2,2-Tetrabromoethane.

**Figure 5:**
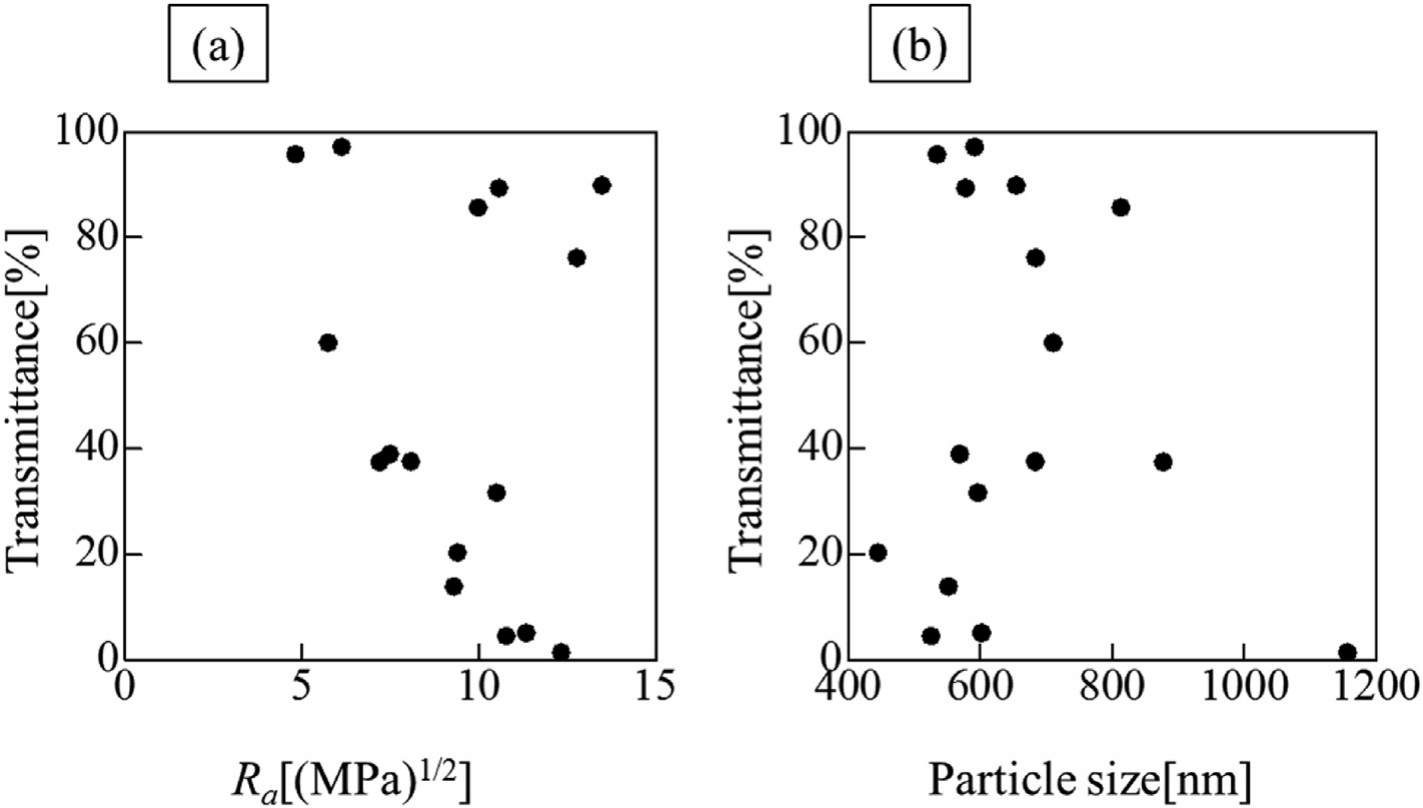
Relationship between transmittance and *R*_*a*_ or particle diameter when light wavelength is 500 nm. (a) Transmittance vs. *R*_*a*_, (b) Transmittance vs. particle size.

As examples of the light transmittance measurements at each wavelength, the results for acetic acid, diethylene glycol monomethyl ether, dimethyl sulfoxide, and 1,1,2,2-tetrabromoethane are presented in Figure 4. The degree of light transmission clearly differed depending on the solvent in which the particles were dispersed. In all the solvents measured, the light transmittance was low in the short-wavelength region. In contrast, the transmittance increased in the long-wavelength region. Shorter light wavelengths corresponded to larger energy and larger incident angles; therefore, the light tended to be scattered. For long light wavelengths, the energy is small and light is not easily scattered. Therefore, the transmittance was considered to decrease with decreasing wavelength. The results of the particle diameter measurement in each solvent and the light transmittance at wavelengths of 400–700 nm are presented in Table 5. The transmission for a light wavelength of 500 nm and HSP difference *R*_*a*_ are plotted in Figure 5a. No correlation was observed transmittance and *R*_*a*_. Figure 5b presents a plot of the particle size and light transmittance in a solvent. No correlation was observed between the particle size and transmittance.

**Table 5:** Relationship between transmittance and difference of *δ*_*d*_.

Material		CEMEDINE C	S-A	S–B
*Δδ*_*d*_ [(MPa)^1/2^]		0.2	0.5	1.8
Transmittance [%]	300 nm	84.5	57.2	25.0
	400 nm	94.7	73.1	43.5
	500 nm	98.0	81.8	57.3
	600 nm	99.3	87.3	67.2
	700 nm	99.9	90.6	73.9

**Figure 6:**
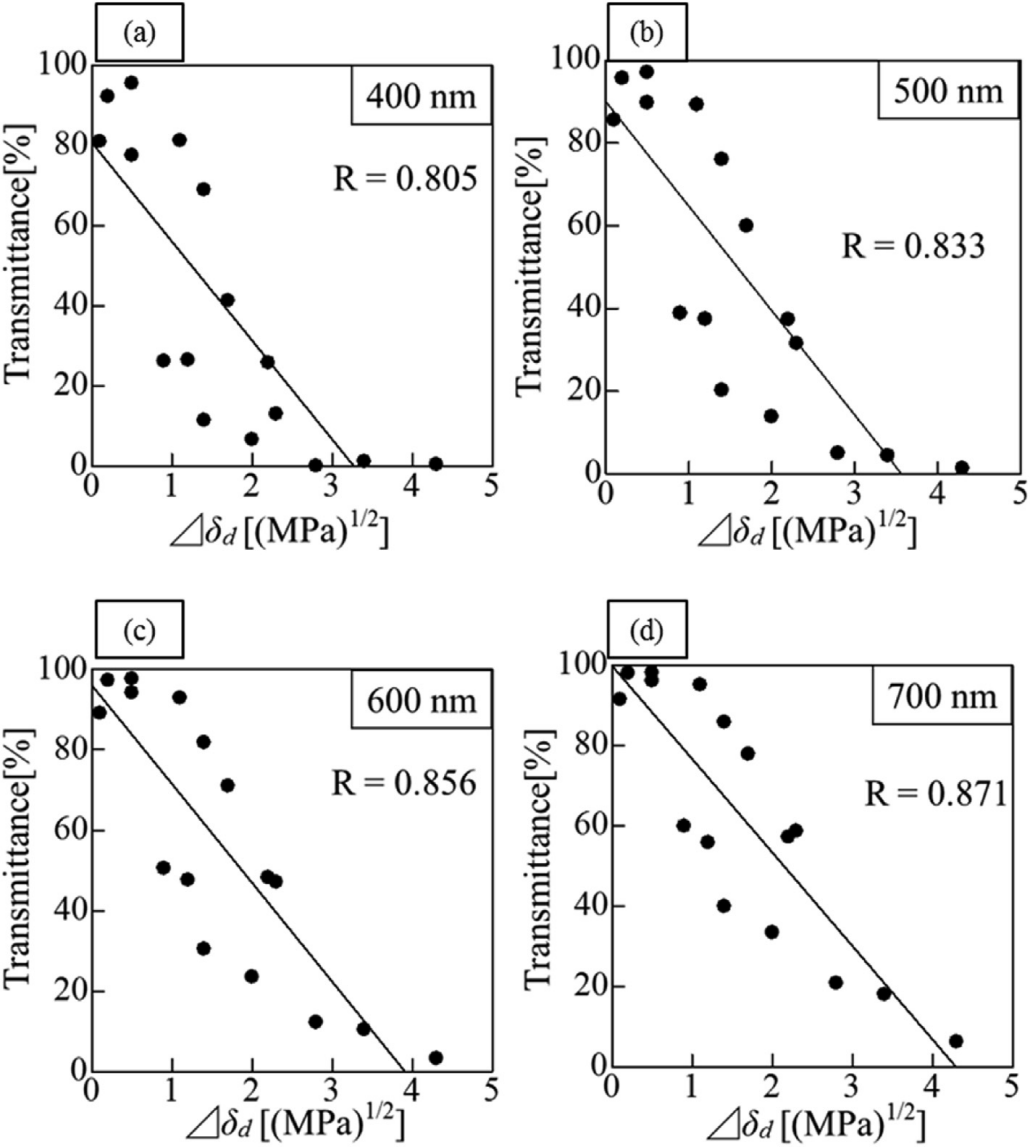
Transmittance of light at each wavelength and difference of *δ*_*d*_ between particle and solvent. (a) Transmittance of light at 400 nm (b) Transmittance of light at 500 nm (c) Transmittance of light at 600 nm (d) Transmittance of light at 700 nm.

**Figure 7:**
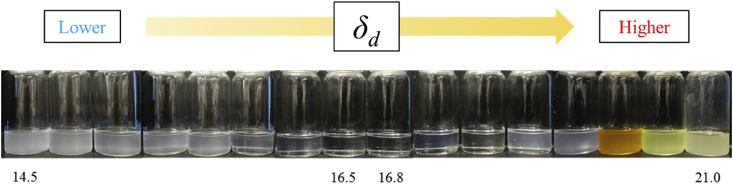
Dispersion state of model particles with various organic solvents.

Figure 6 presents a plot of the difference in of each solvent and particles and the light transmittance. The correlation coefficient between Δ*δ*_*d*_ and the light transmission was R = 0.833. Similarly, the relationship between Δ*δ*_*d*_ and the light wavelength (400, 600, and 700 nm) is shown in Figure 6. A strong correlation with a correlation coefficient R = 0.8 or more was observed in all the systems. Figure 7 shows the dispersion state of each solvent after 5 min of ultrasonic treatment. The samples are arranged in order of decreasing value of *δ*_*d*_ from the left to the right. Visual observation also reveals that *δ*_*d*_ of the solvent became transparent in a certain area and became turbid when outside of certain area. From the above results, it is considered possible to predict and evaluate the light transmittance of the solvent using the difference in *δ*_*d*_ of the HSP. HSP theory was originally used for examining the dispersibility of fine particles and evaluating the solubility of many substances. Therefore, it is considered that the transmittance can be predicted when the optimum dispersion medium for the particles is selected.

### 4.4 Transmittance measurement results for solid-solid systems

**Figure 8:**
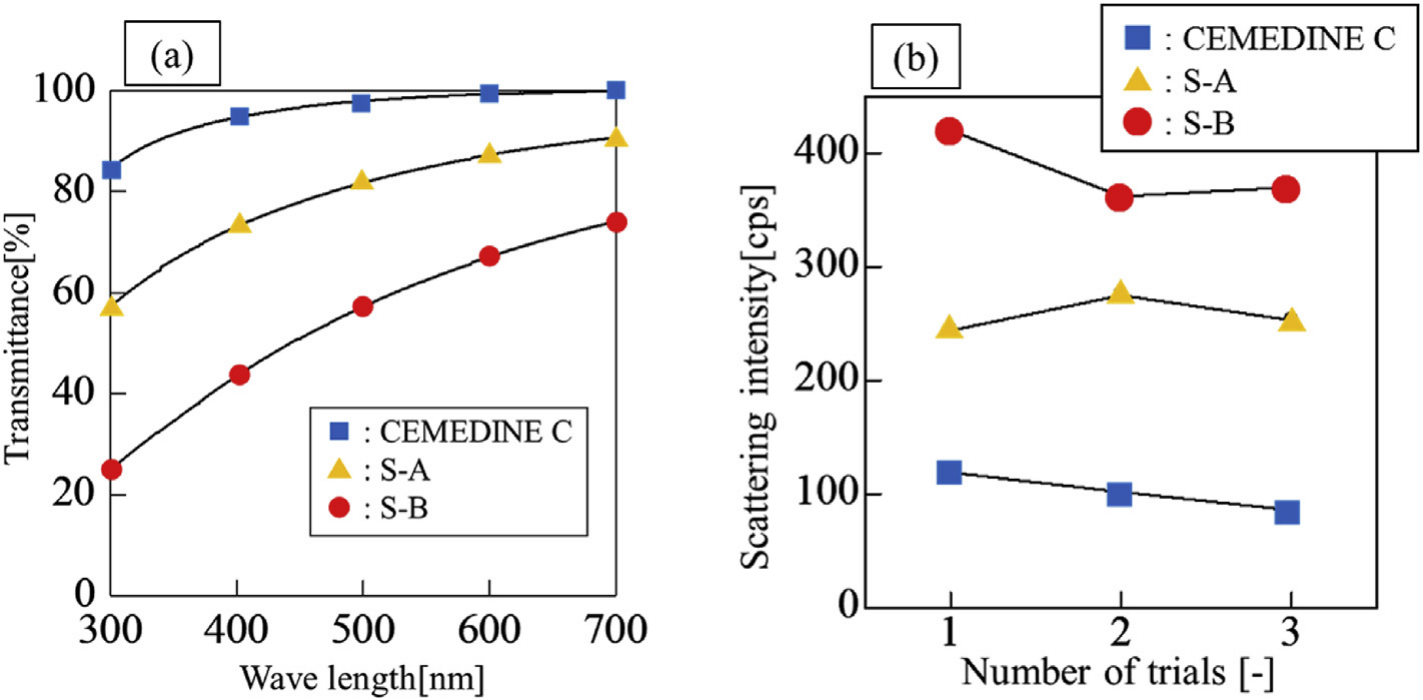
Results of transmissivity measurement for polymer films and Scattering intensity of polymer films. (a) Wave length vs. Transmittance, (b) Scattering intensity date.

The visible light transmittance measurements for the prepared polymer film are presented in Table 5 and Figure 8. A trend of CEMEDINE C > S–A > S–B was observed for the transmittance. Thus, the light transmittance tended to improve with decreasing difference between *δ*_*d*_ of the particle and polymer, as predicted. The scattering intensity measurements for SA, SB, and CEMEDINE C are presented in Figure 8. The scattering intensity cps is an index indicating the degree to which the light incident on the film is scattered. A lower scattering intensity indicates that more light is passed through the film without being scattered. A trend of CEMEDINE C < S–A < S–B was observed in the scattering intensity. The relationship between the mean value of the scattering intensity and ⊿*δ*_*d*_ is shown in Figure 8. A smaller ⊿*δ*_*d*_ was observed to correspond to a smaller scattering intensity.

To obtain sufficient transparency for the specifications required for the polymer film, the value of the refractive index must be controlled to the third decimal place. When considered in conjunction with Eq. (9), it is necessary to control ⊿*δ*_*d*_ to be approximately 0.2 or less. The use of HSP theory has also been suggested to be useful in evaluating the dispersibility of fine particles [5,13]. Therefore, it is considered that the transmittance can be predicted when the optimum dispersion medium for the particles is selected. The results of the current study indicate that the efficiency of both material design and material selection can be improved by using HSP as an index.

## 5 Conclusion

In this study, disc-shaped silica particles in various solvents were examined, and the HSP on the surface of the particles was determined from the contact angle. The transparency was examined using the obtained HSP on the surface of the silica particles. We focused on the value of the *δ*_*d*_ term of that HSP, which has been reported be related to the refractive index. The light transmittance measurements confirmed that this property differed depending on the dispersion medium. A high correlation of R = 0.8 or more between the light transmittance and difference between *δ*_*d*_ of the particle and solvent was confirmed. In the solid–solid verification, the polymer film was prepared, and the transparency evaluation was performed from the two directions of visible light transmittance and scattering intensity measurements. A correlation between ⊿*δ*_*d*_ and the transparency of the model particles and polymer was observed, similar to the results obtained for the solid–liquid system. Our findings demonstrate the possibility of evaluating both the transparency of materials and their dispersibility and compatibility using the same index by applying HSP theory.

## Declarations

### Author contribution statement

Nobuyuki Fujiwara: Conceived and designed the experiments; Performed the experiments; Analyzed and interpreted the data; Contributed reagents, materials, analysis tools or data; Wrote the paper.

Takahiro Nishida: Performed the experiments; Analyzed and interpreted the data; Contributed reagents, materials, analysis tools or data.

Hideki Yamamoto: Performed the experiments; Analyzed and interpreted the data.

### Funding statement

This research did not receive any specific grant from funding agencies in the public, commercial, or not-for-profit sectors.

### Competing interest statement

The authors declare no conflict of interest.

### Additional information

No additional information is available for this paper.

## Appendix A Supplementary data

Supplemental data R2.pptx Supplemental data R2.pptx

## References

[1] Mallakpour S., Barati A. (2011). Efficient preparation of hybrid nanocomposite coatings based on poly(vinyl alcohol) and silane coupling agent modified TiO2 nanoparticles. Prog. Org. Coat..

[2] Paul D.R., Robeson L.M. (2008). Polymer nanotechnology: nanocomposites. Polymer.

[3] Arifin W., Kuboki T. (2018). Effects of glass fibers on mechanical and thermal properties of poly(3-hydroxybutyrate-co-3-hydroxyhexanoate). Polym. Compos..

[4] Granstrom M., Petritsch K., Arias A.C., Lux A., Andersson M.R., Friend R.H. (1998). Laminated fabrication of polymeric photovoltaic diodes. Nature.

[5] Fujiwara N., Imai S., Yamamoto H. (2019). Evaluation of the influence of fine particle surface modification with the Hansen solubility parameters. Mater. Chem. Phys..

[6] Fu S.Y., Lauke B., Mader E., Yue C.Y., Hu X. (2000). Tensile properties of short-glass-fiber- and short-carbon-fiber-reinforced polypropylene composites. Compos. Appl. Sci. Manuf..

[7] Argun A.A., Aubert P.H., Thompson B.C., Schwendeman I., Gaupp C.L., Hwang J., Pinto N.J., Tanner D.B., MacDiarmid A.G., Reynolds J.R. (2004). Multicolored electrochromism polymers: structures and devices. Chem. Mater..

[8] Dang Z.M., Xia Y.J., Zha J.W., Yuan J.K., Bai J.B. (2011). Preparation and dielectric properties of surface modified TiO2/silicone rubber nanocomposites. Mater. Lett..

[9] Tanio N. (2004). Improvement of transparency of optical polymers. Kobunshi Ronbunshu.

[10] Hansen C.M., Smith A.L. (2004). Using Hansen solubility parameters to correlate solubility of C-60 fullerene in organic solvents and in polymers. Carbon.

[11] Hildebrand J.H., Scott R.L. (1950). The Solubility of Nonelectrolytes.

[12] Hansen C.M. (1999). Hansen Solubility Parameters: A User’s Handbook.

[13] Wieneke J.U., Kommoss B., Gaer O., Prykhodko I., Ulbricht M. (2012). Systematic investigation of dispersions of unmodified inorganic nanoparticles in organic solvents with focus on the Hansen solubility parameters. Ind. Eng. Chem. Res..

[14] Fujiwara N., Yamamoto H. (2019). Evaluation of adsorption of organic solvents to modified hydrophobic silica adsorbents based on Hansen solubility parameter. Separ. Purif. Technol..

[15] Araki S., Gondo D., Imasaka S., Yamamoto H. (2016). Permeation properties of organic compounds from aqueous solutions through hydrophobic silica membranes with different functional groups by pervaporation. J. Membr. Sci..

[16] Raynal M., Bouteiller L. (2011). Organogel formation rationalized by Hansen solubility parameters. Chem. Commun..

[17] Novaki L.P., Moraes E.O., Goncalves A.B., de Lira R.A., Linhares V.N., de Oliveira M.C.K., Meireles F.A., Gonzalez G., El Seoud O.A. (2016). Solvatochromic and solubility parameters of solvents: equivalence of the scales and application to probe the solubilization of asphaltenes. Energy Fuel..

[18] Fan X.F., Zheng W.T., Singh D.J. (2014). Light scattering and surface plasmons on small spherical particles. Light Sci. Appl..

[19] Shi J.L., Wu H.P., Yan F., Yang J.J., He X.D. (2016). Experimental study on stimulated scattering of ZnO nanospheres dispersed in water. J. Nanoparticle Res..

[20] Iba H., Kagawa Y. (1998). Light transmittance of continuous fibre-reinforced composites: analysis, model experiment and parametric study. Philos. Mag. B-Physics of Condensed Matter Statistical Mechanics Electronic Optical and Magnetic Properties.

[21] Kinart C.M., Kinart W.J., Kolasinski A. (1998). The internal structures of liquid N-methylformide - N,N-dimethylformamide binary mixtures. Phys. Chem. Liq..

