# Supplementary material for: Adaptation of Hansen solubility parameter in evaluating transparency of composite materials
Source: Heliyon. 2019 Dec 12;5(12):e02833. doi: 10.1016/j.heliyon.2019.e02833 (PMC7019074; doi:10.1016/j.heliyon.2019.e02833)
Supplement: Supplemental data R2.pptx [file mmc1.pptx]

## Slide 1
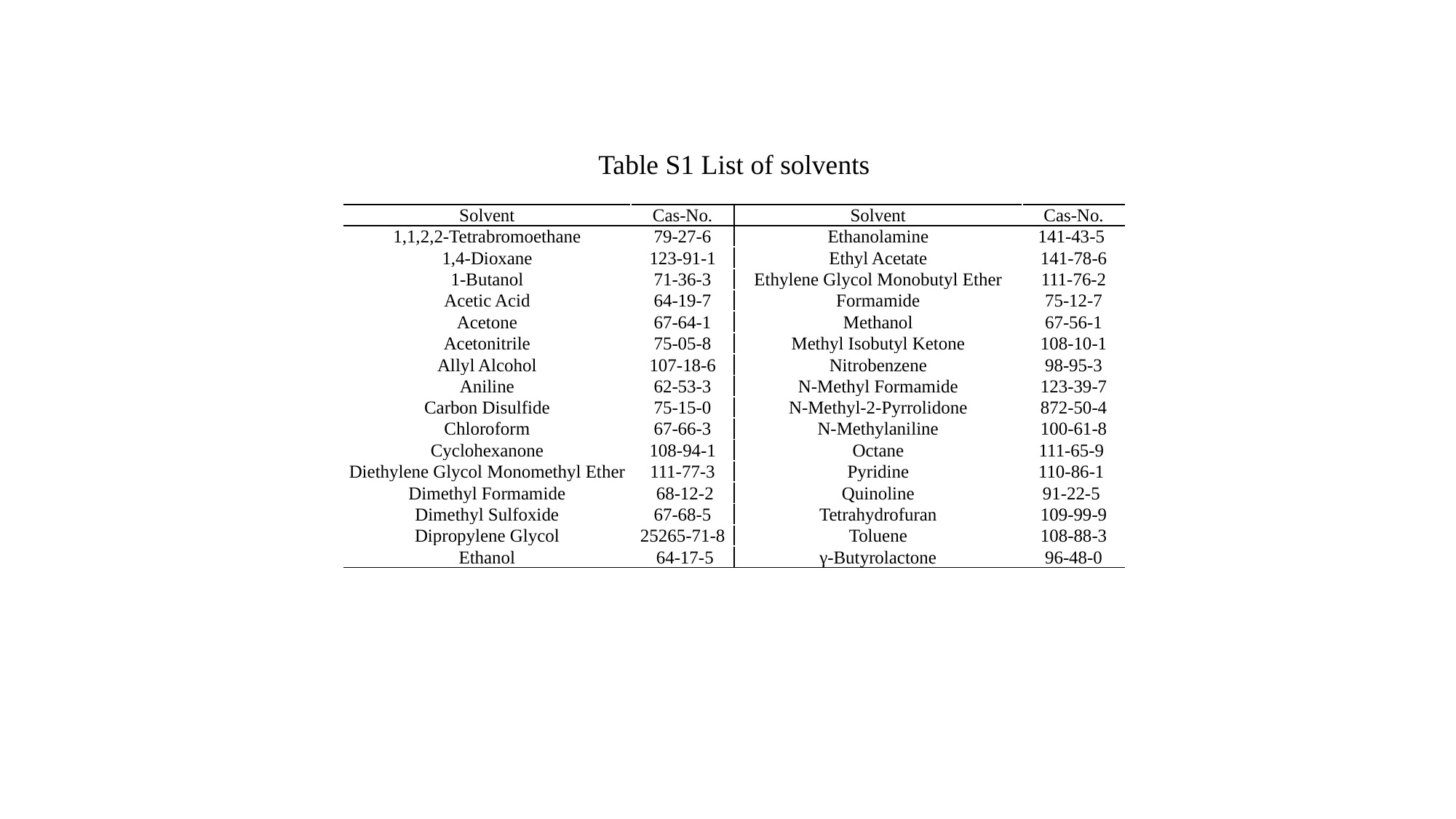

Table S1 List of solvents
| Solvent | Cas-No. | Solvent | Cas-No. |
| --- | --- | --- | --- |
| 1,1,2,2-Tetrabromoethane | 79-27-6 | Ethanolamine | 141-43-5 |
| 1,4-Dioxane | ‎123-91-1 | Ethyl Acetate | 141-78-6 |
| 1-Butanol | 71-36-3 | Ethylene Glycol Monobutyl Ether | 111-76-2 |
| Acetic Acid | 64-19-7 | Formamide | 75-12-7 |
| Acetone | 67-64-1 | Methanol | 67-56-1 |
| Acetonitrile | 75-05-8 | Methyl Isobutyl Ketone | 108-10-1 |
| Allyl Alcohol | 107-18-6 | Nitrobenzene | 98-95-3 |
| Aniline | 62-53-3 | N-Methyl Formamide | 123-39-7 |
| Carbon Disulfide | 75-15-0 | N-Methyl-2-Pyrrolidone | 872-50-4 |
| Chloroform | 67-66-3 | N-Methylaniline | 100-61-8 |
| Cyclohexanone | 108-94-1 | Octane | 111-65-9 |
| Diethylene Glycol Monomethyl Ether | 111-77-3 | Pyridine | 110-86-1 |
| Dimethyl Formamide | 68-12-2 | Quinoline | 91-22-5 |
| Dimethyl Sulfoxide | 67-68-5 | Tetrahydrofuran | 109-99-9 |
| Dipropylene Glycol | 25265-71-8 | Toluene | 108-88-3 |
| Ethanol | 64-17-5 | γ-Butyrolactone | 96-48-0 |

## Slide 2
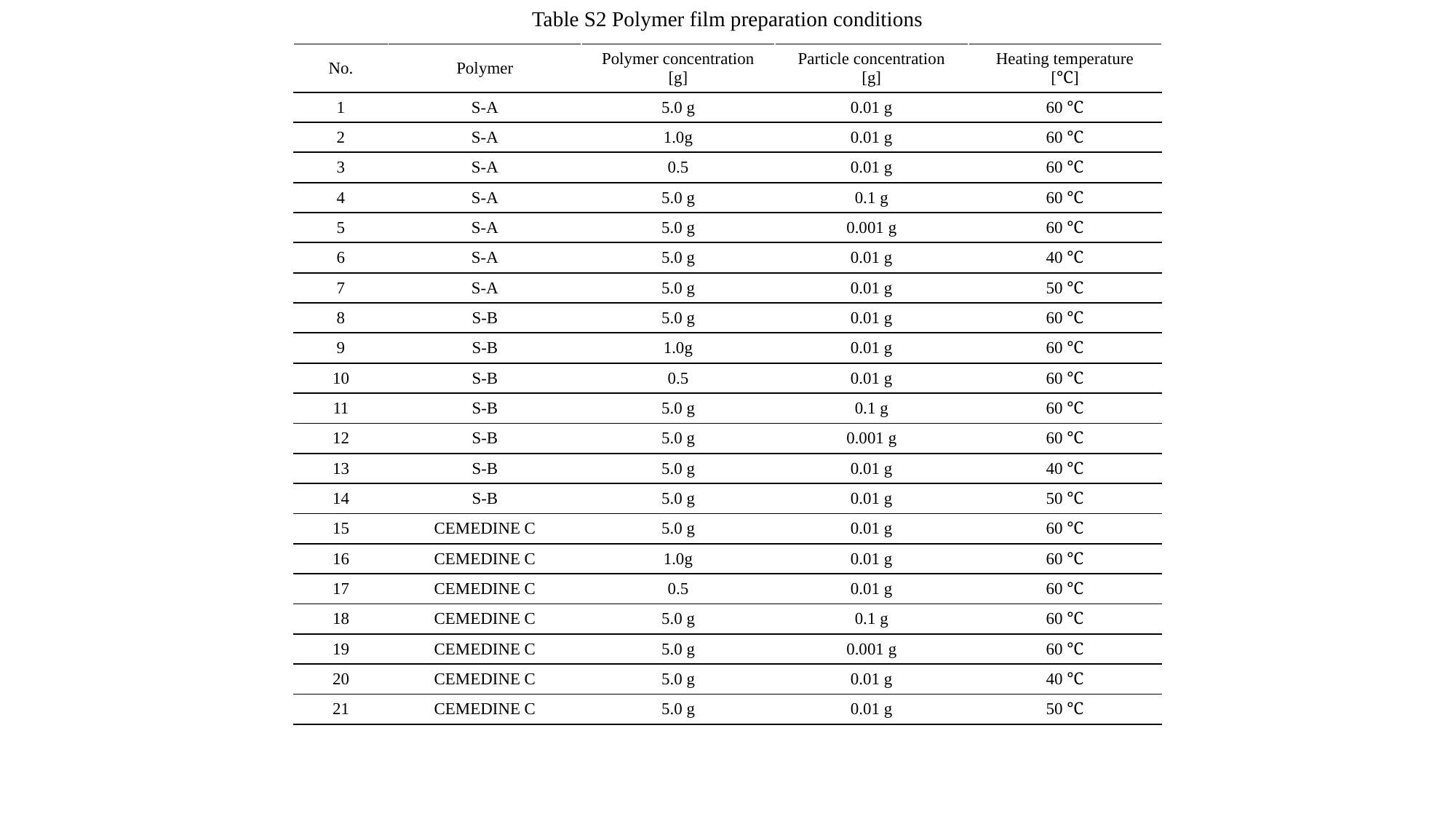

Table S2 Polymer film preparation conditions
| No. | Polymer | Polymer concentration [g] | Particle concentration [g] | Heating temperature [℃] |
| --- | --- | --- | --- | --- |
| 1 | S-A | 5.0 g | 0.01 g | 60 ℃ |
| 2 | S-A | 1.0g | 0.01 g | 60 ℃ |
| 3 | S-A | 0.5 | 0.01 g | 60 ℃ |
| 4 | S-A | 5.0 g | 0.1 g | 60 ℃ |
| 5 | S-A | 5.0 g | 0.001 g | 60 ℃ |
| 6 | S-A | 5.0 g | 0.01 g | 40 ℃ |
| 7 | S-A | 5.0 g | 0.01 g | 50 ℃ |
| 8 | S-B | 5.0 g | 0.01 g | 60 ℃ |
| 9 | S-B | 1.0g | 0.01 g | 60 ℃ |
| 10 | S-B | 0.5 | 0.01 g | 60 ℃ |
| 11 | S-B | 5.0 g | 0.1 g | 60 ℃ |
| 12 | S-B | 5.0 g | 0.001 g | 60 ℃ |
| 13 | S-B | 5.0 g | 0.01 g | 40 ℃ |
| 14 | S-B | 5.0 g | 0.01 g | 50 ℃ |
| 15 | CEMEDINE C | 5.0 g | 0.01 g | 60 ℃ |
| 16 | CEMEDINE C | 1.0g | 0.01 g | 60 ℃ |
| 17 | CEMEDINE C | 0.5 | 0.01 g | 60 ℃ |
| 18 | CEMEDINE C | 5.0 g | 0.1 g | 60 ℃ |
| 19 | CEMEDINE C | 5.0 g | 0.001 g | 60 ℃ |
| 20 | CEMEDINE C | 5.0 g | 0.01 g | 40 ℃ |
| 21 | CEMEDINE C | 5.0 g | 0.01 g | 50 ℃ |

## Slide 3
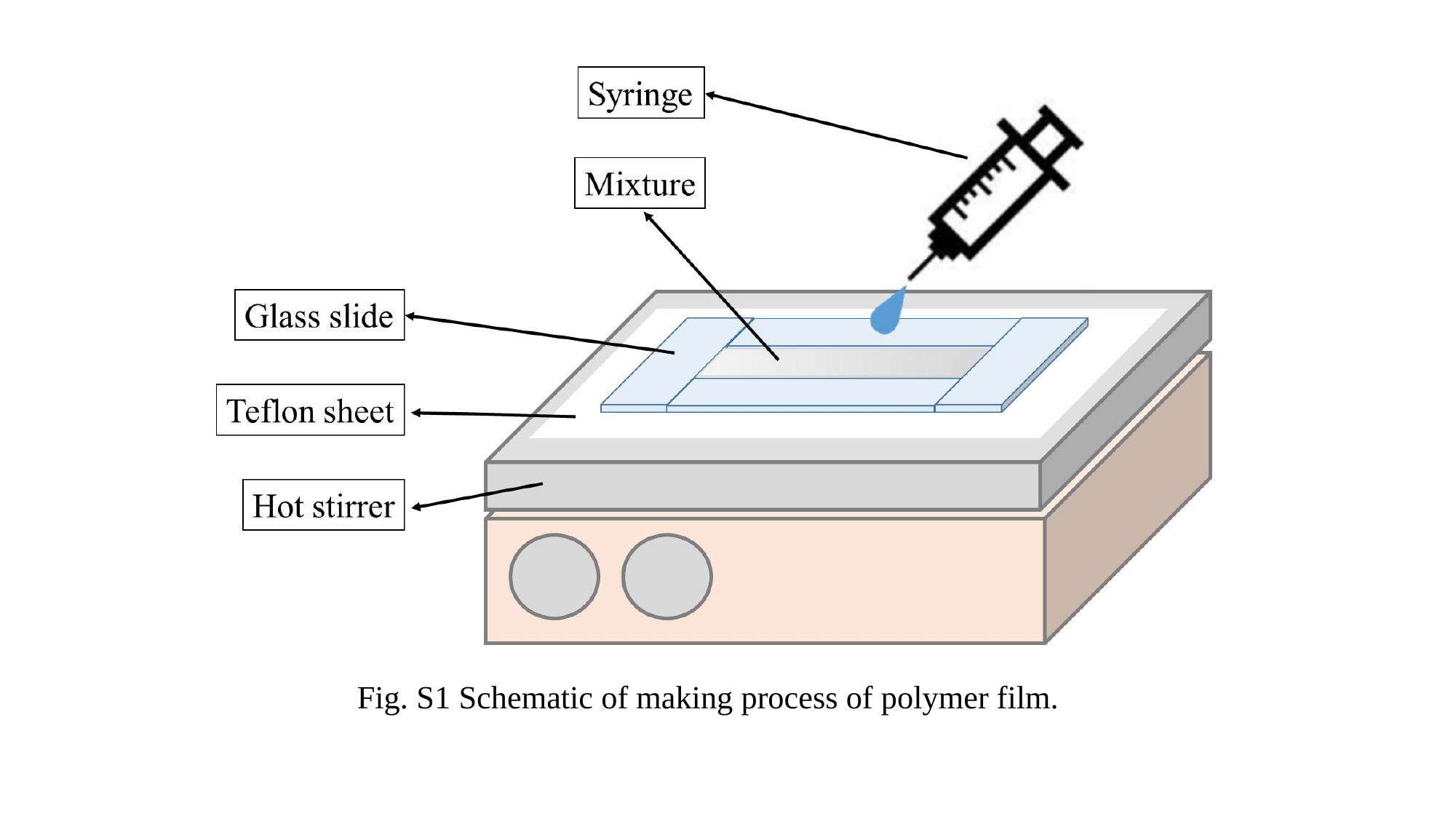

Fig. S1 Schematic of making process of polymer film.

## Slide 4
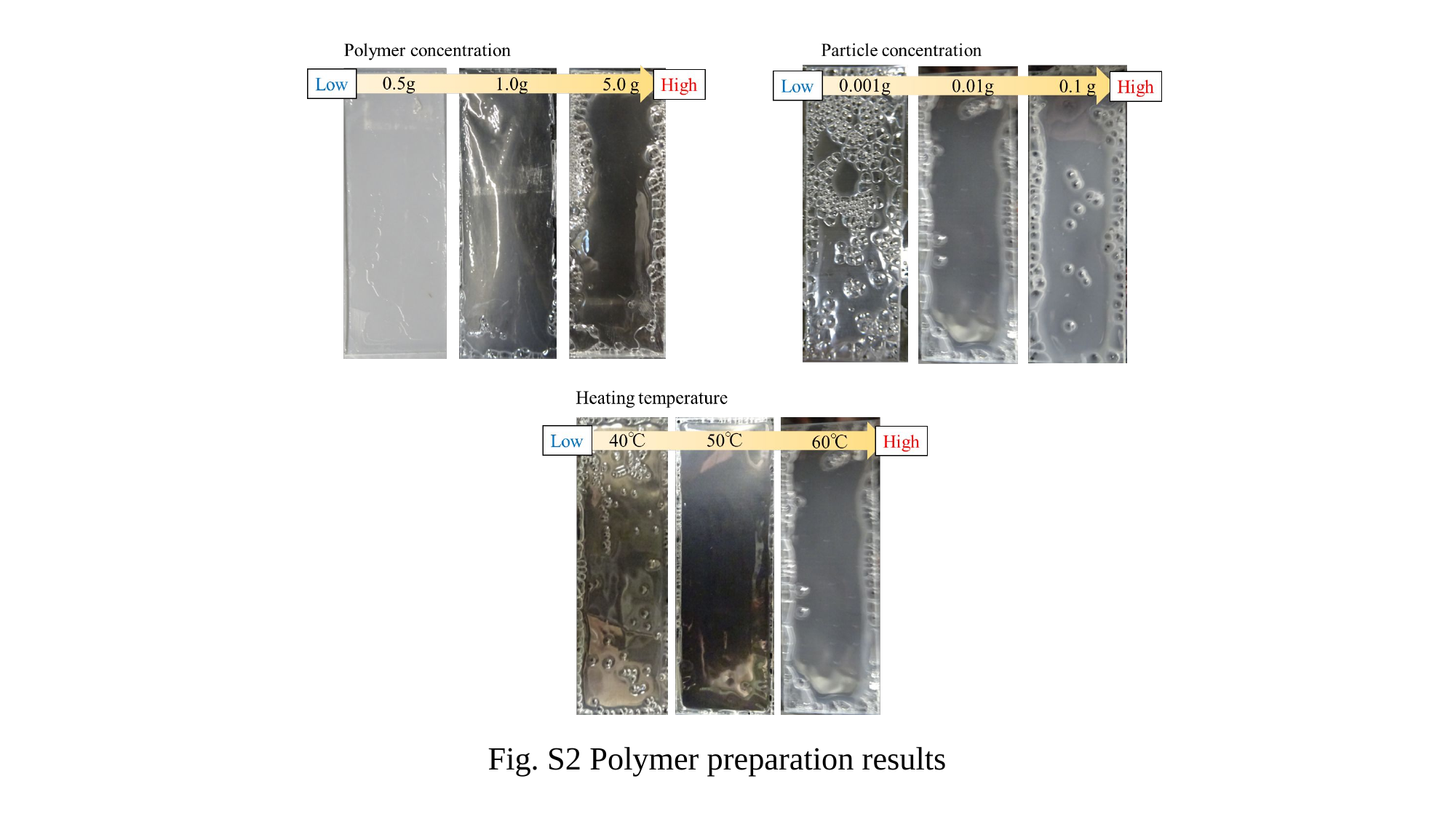

Fig. S2 Polymer preparation results
